# Supplementary material for: Predictors of infection, symptoms development, and mortality in people with SARS-CoV-2 living in retirement nursing homes
Source: PLoS One. 2021 Mar 16;16(3):e0248009. doi: 10.1371/journal.pone.0248009 (PMC7963051; doi:10.1371/journal.pone.0248009)
Supplement: S1 Table — (DOCX) [file pone.0248009.s001.docx]

|  | **Unadjusted** | | **Adjusted** | |
| --- | --- | --- | --- | --- |
|  | **Odds ratio (95% CI)** | *p-value* | **Odds ratio (95% CI)** | *p-value* |
| **Gender** |  |  |  |  |
| Female vs Male | 1.13 (0.72-1.77) | 0.606 |  |  |
| **Comorbidities** |  |  |  |  |
| BMI > 30 | 1.40 (0.78-2.52) | 0.258 |  |  |
| Hypertension | 1.34 (0.87-2.08) | 0.187 | 0.96 (0.59-1.58) | 0.881 |
| Diabetes | 1.85 (0.998-3.43) | 0.051 | 1.43 (0.74-2.75) | 0.286 |
| COPD | 1.53 (0.84-2.77) | 0.162 | 1.27 (0.67-2.39) | 0.461 |
| CHD | 1.61 (1.01-2.60) | 0.049 | 1.63 (0.97-2.75) | 0.064 |
| Mental illness | 1.85 (1.17-2.91) | 0.008 | 1.81 (1.13-2.91) | 0.013 |
| Neurological | 0.57 (0.37-0.89) | 0.013 | 0.61 (0.38-0.98) | 0.041 |
| Kidney failure | 1.13 (0.48-2.64) | 0.783 |  |  |
| Cancer | 6.34 (1.48 – 27.15) | 0.013 | 6.37(1.45-28.03) | 0.014 |
| Compliance | 1.53 (0.99-2.37) | 0.058 | 1.16 (0.72-1.88) | 0.543 |
| Hypokinetic disease | 1.30 (0.78-2.17) | 0.314 |  |  |
| **Chronic Treatment** |  |  |  |  |
| ARBs | 2.75 (1.25-6.04) | 0.012 | 2.40(1.04-5.54) | 0.04 |
| ACE inhibitor | 0.84 (0.51-1.41) | 0.519 |  |  |
| CI: confidence interval; BMI: body mass index; COPD: chronic obstructive pulmonary disease; CHD: cardiovascular disease; ARBs: Angiotensin II receptor blockers; ACE: angiotensin-converting enzyme. | | | | |

S1 Table. Bivariate and multivariate logistic regression estimates of factors associated with SARS-CoV-2 infection.
