# Supplementary material for: Predictors of infection, symptoms development, and mortality in people with SARS-CoV-2 living in retirement nursing homes
Source: PLoS One. 2021 Mar 16;16(3):e0248009. doi: 10.1371/journal.pone.0248009 (PMC7963051; doi:10.1371/journal.pone.0248009)
Supplement: S2 Table — (DOCX) [file pone.0248009.s002.docx]

|  | **Unadjusted** | | **Adjusted** | |
| --- | --- | --- | --- | --- |
|  | **Odds ratio (95% CI)** | *p-value* | **Odds ratio (95% CI)** | *p-value* |
| **Gender** |  |  |  |  |
| Female vs Male | 1.34 (0.81-2.21) | 0.253 |  |  |
| **Comorbidities** |  |  |  |  |
| BMI > 30 | 1.26 (0.69-2.29) | 0.461 |  |  |
| Hypertension | 1.47 (0.89-2.41) | 0.131 | 1.16 (0.66-2.02) | 0.603 |
| Diabetes | 0.76 (0.42-1.38) | 0.367 |  |  |
| COPD | 2.18 (1.19-4.01) | 0.012 | 1.96 (1.04-3.70) | 0.037 |
| CHD | 1.74 (1.05-2.88) | 0.031 | 1.35 (0.78-2.33) | 0.28 |
| Mental illness | 0.89 (0.55-1.44) | 0.622 |  |  |
| Neurological | 1.86 (1.13-3.03) | 0.014 | 1.8 (1.07-3.01) | 0.026 |
| Kidney failure | 1.95 (0.75-5.05) | 0.169 |  |  |
| Cancer | 0.84 (0.37-1.90) | 0.680 |  |  |
| Hypokinetic disease | 1.53 (0.88-2.65) | 0.128 | 1.3 (0.72-2.35) | 0.384 |
| **Chronic Treatment** |  |  |  |  |
| ARBs | 1.82 (0.95-3.51) | 0.074 | 1.72 (0.84-3.52) | 0.14 |
| ACE inhibitor | 1.05 (0.58-1.88) | 0.881 |  |  |
| CI: confidence interval; BMI: body mass index; COPD: chronic obstructive pulmonary disease; CHD: cardiovascular disease; ARBs: Angiotensin II receptor blockers; ACE: angiotensin-converting enzyme. | | | | |

S2 Table. Bivariate and multivariate logistic regression estimates of factors associated with SARS-CoV-2-related symptoms.
