# Supplementary material for: Predictors of infection, symptoms development, and mortality in people with SARS-CoV-2 living in retirement nursing homes
Source: PLoS One. 2021 Mar 16;16(3):e0248009. doi: 10.1371/journal.pone.0248009 (PMC7963051; doi:10.1371/journal.pone.0248009)
Supplement: S3 Table — (DOCX) [file pone.0248009.s003.docx]

|  | **Unadjusted** | | **Adjusted** | |
| --- | --- | --- | --- | --- |
|  | **Hazard ratio (95% CI)** | *p-value* | **Hazard ratio (95% CI)** | *p-value* |
| **Gender** |  |  |  |  |
| Male vs Female | 1.69 (0.97-2.94) | 0.064 | 1.93 (1.07-3.45) | 0.028 |
| **Comorbidities** |  |  |  |  |
| BMI > 30 | 1.07 (0.53-2.15) | 0.848 |  |  |
| Hypertension | 0.66 (0.38-1.16) | 0.148 | 0.74 (0.42-1.32) | 0.310 |
| Diabetes | 1.55 (0.84-2.88) | 0.163 | 1.48 (0.77-2.84) | 0.240 |
| COPD | 1.81 (0.997-3.27) | 0.051 | 0.85 (0.43-1.67) | 0.631 |
| CHD | 1.36 (0.77-2.37) | 0.285 |  |  |
| Mental illness | 0.83 (0.47-1.44) | 0.501 |  |  |
| Neurological | 1.80 (1.03-3.17) | 0.039 | 1.09 (0.61-1.95) | 0.783 |
| Kidney failure | 1.11 (0.40-3.08) | 0.844 |  |  |
| Cancer | 0.78 (0.28-2.16) | 0.632 |  |  |
| Hypokinetic disease | 4.05 (2.31-7.08) | <0.001 | 1.91 (1.05-3.48) | 0.035 |
| Autonomy | 0.039 (0.005-0.28) | 0.001 | 0.051 (0.007-0.39) | 0.004 |
| **Symptomatology** |  |  |  |  |
| Fever + Dyspnea | 3.79 (2.17-6.61) | <0.001 | 3.99 (2.05-7.79) | <0.001 |
| **Chronic Treatment** |  |  |  |  |
| ARBs | 1.43 (0.73-2.78) | 0.561 |  |  |
| ACE inhibitor | 0.77 (0.38-1.60) | 0.495 |  |  |
| **SARS-COV-2 Treatment** |  |  |  |  |
| Hydroxychloroquine | 0.97 (0.5-1.9) | 0.937 |  |  |
| Azithromycin | 0.82 (0.39-1.75) | 0.615 |  |  |
| LMWH | 0.61 (0.33-1.11) | 0.106 | 0.42 (0.22-0.79) | 0.008 |
| CI: confidence interval; BMI: body mass index; COPD: chronic obstructive pulmonary disease; CHD: cardiovascular disease; ARBs: Angiotensin II receptor blockers; ACE: angiotensin-converting enzyme. LMWH: low molecular weight heparin | | | | |

S3 Table. Bivariate and multivariate Cox proportional-hazards model estimates of factors associated with mortality.
