# Supplementary figures and images for: Predictors of infection, symptoms development, and mortality in people with SARS-CoV-2 living in retirement nursing homes
Source: PLoS One. 2021 Mar 16;16(3):e0248009. doi: 10.1371/journal.pone.0248009 (PMC7963051; doi:10.1371/journal.pone.0248009)

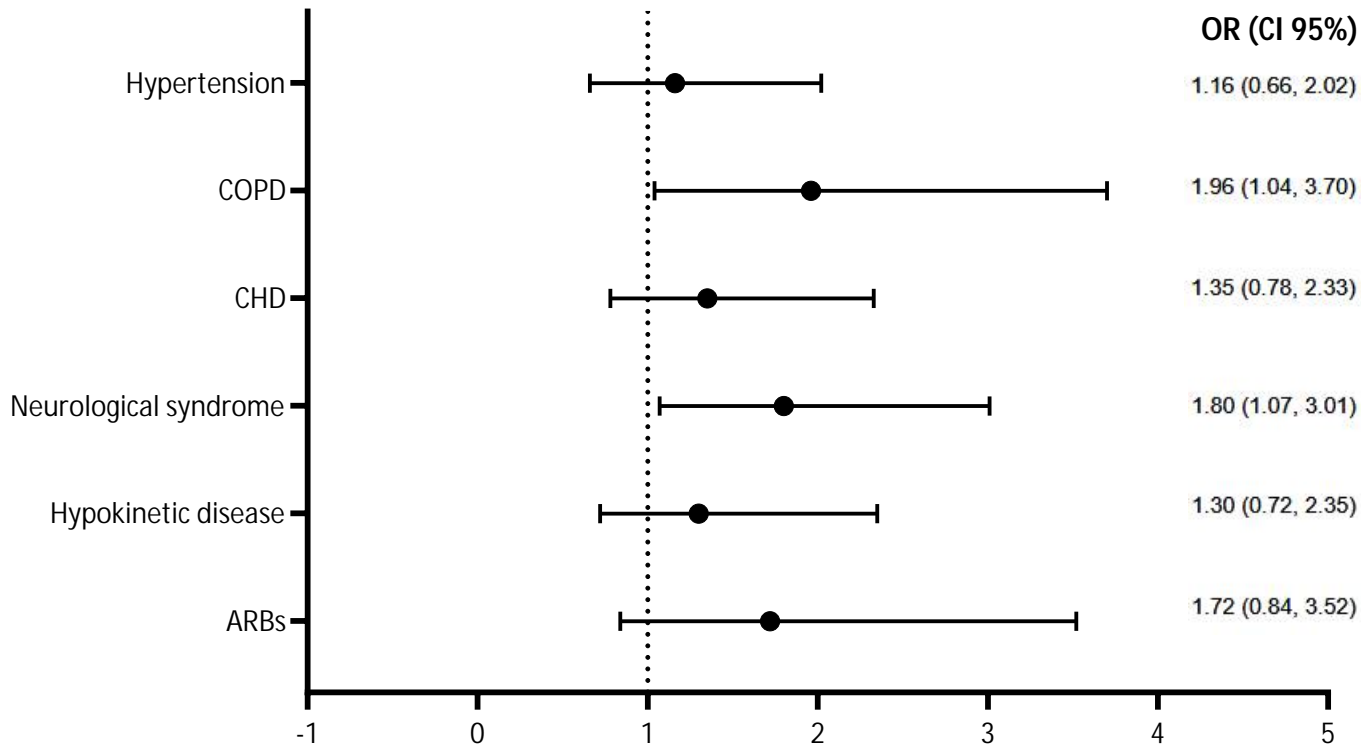

Supplement: S1 Fig — Multivariable model includes all variables selected by a backward selection that were retained with a p-value less than 0.2 level. OR: Odds Ratio; CI: Confidence Interval; COPD: chronic obstructive pulmonary disease; CHD: cardiovascular disease; ARBs: Angiotensin II receptor blockers. (PDF) [file pone.0248009.s004.pdf]
