# Supplementary material for: Predictors of infection, symptoms development, and mortality in people with SARS-CoV-2 living in retirement nursing homes
Source: PLoS One. 2021 Mar 16;16(3):e0248009. doi: 10.1371/journal.pone.0248009 (PMC7963051; doi:10.1371/journal.pone.0248009)
Supplement: Attachment — Submitted filename: Authors reply R2.docx [file pone.0248009.s008.docx]

Dear Professor Calcagno,

I would like to thank you for allowing us to reply to reviewer n°2.

Reviewer concern:

Second, there is no need to say anything about the 'established p-values' as the authors show the exact p-value instead of using Asterix (which is the right thing to do).

However, there is a mention that only the variable where p<.2 where included. This is ad hoc and needs a rationale. I am still unsure what it means and what happened, and it should be justified."

Authors reply:

Thank you for your comment. We included all variables in the multivariate model with a p-value <0.2 because we preferred to be conservative. Otherwise, we could have decided to use a less conservative model, including only variables with a p-value <0.1 or 0.15, but this way, there could have been the possibility to exclude some clinically important variables or adjustments.

Regarding the choice to specify that we considered the p-value statistically significant when <0.05, we preferred to do so to make it as clear as possible to the reader.

Yours sincerely,

Andrea De Vito
